# Iba-1^-^/CD68^+^ microglia are a prominent feature of age-associated deep subcortical white matter lesions

**DOI:** 10.1371/journal.pone.0210888

**Published:** 2019-01-25

**Authors:** Rachel Waller, Lynne Baxter, Daniel J. Fillingham, Santiago Coelho, Jose M. Pozo, Meghdoot Mozumder, Alejandro F. Frangi, Paul G. Ince, Julie E. Simpson, J. Robin Highley

**Affiliations:** 1 Department of Neuroscience, Sheffield Institute for Translational Neuroscience, The University of Sheffield, Sheffield, South Yorkshire, United Kingdom; 2 School of Computing, Center for Computational Imaging & Simulation Technologies in Biomedicine, The University of Leeds, Leeds, West Yorkshire, United Kingdom; 3 Department of Engineering, Center for Computational Imaging & Simulation Technologies in Biomedicine, The University of Sheffield, Sheffield, South Yorkshire, United Kingdom; University of Modena and Reggio Emilia, ITALY

## Abstract

Deep subcortical lesions (DSCL) of the brain, are present in ~60% of the ageing population, and are linked to cognitive decline and depression. DSCL are associated with demyelination, blood brain barrier (BBB) dysfunction, and microgliosis. Microglia are the main immune cell of the brain. Under physiological conditions microglia have a ramified morphology, and react to pathology with a change to a more rounded morphology as well as showing protein expression alterations. This study builds on previous characterisations of DSCL and radiologically ‘normal-appearing’ white matter (NAWM) by performing a detailed characterisation of a range of microglial markers in addition to markers of vascular integrity. The Cognitive Function and Ageing Study (CFAS) provided control white matter (WM), NAWM and DSCL human post mortem tissue for immunohistochemistry using microglial markers (Iba-1, CD68 and MHCII), a vascular basement membrane marker (collagen IV) and markers of BBB integrity (fibrinogen and aquaporin 4). The immunoreactive profile of CD68 increased in a stepwise manner from control WM to NAWM to DSCL. This correlated with a shift from small, ramified cells, to larger, more rounded microglia. While there was greater Iba-1 immunoreactivity in NAWM compared to controls, in DSCL, Iba-1 levels were reduced to control levels. A prominent feature of these DSCL was a population of Iba-1^-^/CD68^+^ microglia. There were increases in collagen IV, but no change in BBB integrity. Overall the study shows significant differences in the immunoreactive profile of microglial markers. Whether this is a cause or effect of lesion development remains to be elucidated. Identifying microglia subpopulations based on their morphology and molecular markers may ultimately help decipher their function and role in neurodegeneration. Furthermore, this study demonstrates that Iba-1 is not a pan-microglial marker, and that a combination of several microglial markers is required to fully characterise the microglial phenotype.

## Introduction

T2-weighted magnetic resonance image (MRI) white matter hyperintensities are a common feature of the ageing brain [1]. These white matter lesions (WML) are classified based on their anatomical location, with periventricular lesions (PVL) found in white matter (WM) next to ventricles, while deep subcortical lesions (DSCL) occur within the centrum semiovale. DSCL are found in around 60% of the population over 65 years of age and are linked to progressive cognitive decline and depression [2]. The definitive cause(s) of WML are, as yet, unknown, yet there is much evidence to suggest blood brain barrier (BBB) dysfunction [3], axonal damage [4, 5] and cerebral hypoperfusion [6] contribute to their pathogenesis.

WML are associated with myelin loss, BBB dysfunction, and an increase in reactive glia including the presence of swollen, fibrinogen^+^ clasmatodendritic astrocytes, alongside an increase in microglial response [7]. Microglia are the main immune cell of the central nervous system (CNS), capable of expressing a variety of cytokines and chemokines that are responsible for the cell’s function. In general, microglia are classed as M1, a ‘pro-inflammatory’ phenotype and M2, an ‘anti-inflammatory’ phenotype, these distinct roles have been investigated and challenged profusely in the literature and it is apparent that this dichotomy is not as clear-cut as initially suggested [8, 9].

Under physiological conditions microglia appear resting and ramified with extending processes constantly sensing the brain environment for any change or pathology that can cause them to respond in a variety of different ways. Cell morphology alone does not fully indicate microglial function and it is therefore important to examine a range of microglial markers to gain a better understanding of these cells in the DSCL and the surrounding WM environment in the ageing brain [10, 11]. Such studies reveal that the microglial response in both PVL and DSCL differs considerably. PVL contain high levels of activated microglia expressing major histocompatibility complex class II (MHCII) and the costimulatory molecules B7-2 and CD40, along with minichromosome maintenance protein 2 (MCM-2) suggesting a more immune-activated and proliferative-permissive environment within PVL [12]. In contrast, DSCL contain low levels of MHCII^+^ microglia instead, high levels of CD68^+^ microglia with amoeboid morphology are present. CD68 is a transmembrane glycoprotein protein expressed by human monocytes and tissue macrophages that indicates phagocytic activity, suggesting that within DSCL phagocytic microglia are likely to be involved in the removal of degraded myelin [12]. The distinct differences in microglial phenotype suggest that the nature of the microglial response differs between DSCL and PVL.

Ionized calcium-binding adaptor molecule 1 (Iba-1), a cytoplasmic protein, is considered a pan microglial marker [13]. While several studies have demonstrated its expression correlates with microglial activation and inflammation [10, 14, 15], no studies have determined Iba-1 expression in age-associated WML.

The current study builds on the previous histological characterisation of age-associated WML and compares the detailed immunoreactive profile of three classic microglial markers, namely Iba-1, CD68 and MHCII. In addition, this study highlights the correlation between two independent image analytic methods of quantifying these microglial markers in the well characterised ageing population-representative Cognitive Function and Ageing Study (CFAS) neuropathology cohort. Furthermore, the study assesses whether changes in vascular integrity are associated with changes in microglial responses and the implication this may have for the pathogenesis of age-associated DSCL.

## Materials and methods

### Tissue

Human autopsy CNS tissue was obtained from the CFAS neuropathology cohort, a well characterised large-prospective multi-centre, ageing population-representative based UK study [16]. Informed written consent was obtained from all participants during life. All CFAS participants when approached are assessed for mental capacity by research interviewers who are fully trained in the mental capacity act. Where it is felt a participant no longer has capacity to consent for themselves a consultee (nominated by the participants previously), is approached and asked whether in their opinion the participant would have wished to continue to take part in the study if they had capacity. Where the consultee agrees that the participant would wish to continue as part of the study we request the consultee sign a declaration agreeing for the participant to continue to take part in the study. For brain donation where someone no longer has the capacity to consent for themselves we get a signed consultee assent to brain donation which details how they feel the participant would have wished the brain tissue to be used. The South Birmingham Research Ethics Committee approved the project (REC Ref: 16/WM/0187; IRAS Project ID: 19826). Anatomically-defined formalin-fixed post-mortem coronal brain slices underwent MRI to assess WM pathology and the tissue sampled, as previously described [1–3]. In brief, blocks were sampled from three coronal slices of approximately 1cm thickness from the level of: the head of the caudate nucleus (anterior slice); the lateral geniculate nucleus (middle slice) and tip of occipital horn of the lateral ventricle (posterior slice). The MR images of each brain slice were rated by two experienced radiologists (blind to clinical status) and given a score for DSCL using a modified Scheltens’ scale [17]. Using these Scheltens’ scores and the MRI images, 3 block types were sampled from WM by experienced neuropathologists for histological analysis, corresponding to the “nested study” design of WML using this tissue resource [1–3]. Control blocks of WM were taken from cases where all three coronal slices were scored as 0. DSCL blocks were taken from regions with a score of 5 or greater. NAWM blocks were taken from lesion free regions of WM in which a DSCL of score 3 or greater was present elsewhere (Table 1). Each tissue block was then subjected to pathological assessment to confirm classification as control, NAWM or DSCL.

**Table 1:** Age, sex, post mortem delay (PMD) of CFAS brain donors.

	Median age (years) (min-max)	Sex (M/F)	PMD (h) (min-max)*
**Control** (18 blocks from 18 non-lesional brains)	87 (71–96)	7/11	43 (7–120)
**NAWM** (16 blocks from 16 individuals)	86 (73–96)	6/10	48 (25–96)
**DSCL** (20 blocks from 20 individuals)	86 (71–101)	10/10	42 (17–164)

*Information not available for 28 individuals.

Key: CFAS, Cognitive Function and Ageing Study; M, male; F, female; NAWM, normal appearing white matter; DSCL, deep subcortical lesion; h, hours

### Immunohistochemistry

Formalin-fixed, paraffin-embedded (FFPE) blocks were sectioned at 5μm, deparaffinised and subjected to antigen retrieval. Sections were then immunostained for Iba-1, CD68 and MHCII (microglia), glial fibrillary acidic protein (GFAP, astrocytes), proteolipid protein (PLP, myelin), collagen IV (Coll IV, basal lamina), fibrinogen (a serum protein marker) and aquaporin 4 (AQP4, a water channel marker) using an intelliPATH FLX system (A. Menarini Diagnostics Ltd, Winnersh, UK). A summary of all the primary antibodies and their conditions of use is shown in Table 2. IHC was performed using a standard IntelliPATH FLX Polymer Detection Kit, visualised using diaminobenzidine tetrachloride (DAB) and sections counterstained with haematoxylin.

**Table 2:** Antibody source, specificity and dilution.

Antibody	Isotype	Dilution (time, temp)	Antigen retrieval method	Supplier
Iba-1	Mouse IgG1κ	1:100 (30 min, RT) or (dual IHC 1hr, RT)	Menarini Diagnostics, Access Revelation, pH6.4, PC	Millipore, MABN92
CD68	Mouse IgG3	1:100 (30 min, RT), or (dual IHC o/n, 4°C)	Menarini Diagnostics, Access Revelation, pH6.4, PC	Abcam, PG-M1, ab783
MHCII	Mouse IgG1κ	1:50 (30 min, RT)	Menarini Diagnostics, Access Revelation, pH6.4, PC	Dako, M0746
GFAP	Rabbit IgG	1:2000 (30 min, RT)	Menarini Diagnostics, Access Revelation, pH6.4, PC	Dako, Z0334
PLP	Mouse IgG2a	1:800 (30 min, RT)	Menarini Diagnostics, Access Revelation, pH6.4, PC	BioRad, MCA839G
Collagen IV (Coll IV)	Rabbit IgG	1:500 (30 min, RT)	Menarini Diagnostics, Access Revelation, pH6.4, PC	Abcam, ab6586
Fibrinogen	Rabbit Ig fraction	1:1000 (30 min, RT)	Menarini Diagnostics, Access Revelation, pH6.4, PC	Dako, A0080
Aquaporin 4 (AQP4)	Rabbit IgG	1:400 (30 min, RT)	Menarini Diagnostics, Access Revelation, pH6.4, PC	Thermo Fisher Scientific, PA5-36521

Key: MHCII, major histocompatibility complex II; GFAP, glial fibrillary acidic protein; PLP, Proteolipid protein; PC, pressure cooker.

The immunoreactive profile of microglia within the DSCL was further assessed by dual labelling with Iba-1 and CD68 and was completed manually. Following Iba-1 antibody labelling using a standard horse-radish peroxidase avidin-biotin complex (ABC) method with DAB as substrate (Vector Laboratories, UK) as outline above, Iba-1 stained sections were incubated with the avidin-biotin blocking kit (Vector Laboratories, UK), and incubated overnight at 4°C with anti-CD68, followed by the alkaline-phosphatase-conjugated ABC (Vectastain Elite kit, Vector Laboratories, UK), developed with alkaline phosphatase substrate (Vector Laboratories, UK; red) and counterstained with haematoxylin. Additional dual labelling was completed where Iba-1 stained sections (using a standard ABC-HRP method as earlier) were double labelled, respectively, with CD68 using Streptavidin Alexa Fluor 555 conjugate (Life Technologies, UK) at 1:500 dilution in replace of the ABC and DAB substrate. Negative controls consisted of sections incubated with isotype controls or in the absence of primary antibody.

### Digital histology and image analysis

#### Quantitative analysis

All immunostained sections were digitally scanned under a 40x objective lens using a Nanozoomer XR (Hamamatsu, Photonics Ltd., Hertfordshire, UK). The final resolution of these images was of 0.23μm/pixel. Scanned sections were stored as NanoZoomer Digital Pathology Image (.ndpi) files, and viewed using NDP.View 2 and analysed in Visiopharm and/or with an in-house toolbox developed in MATLAB (The Mathworks, Natick, MA). Additionally a semi-quantitative analysis was completed on the fibrinogen-labelled sections. WM regions of interest (ROI) were selected based on visually assessing the extent of microglial activation. In Visiopharm the ROI was depicted through Iba-1 and CD68 immunoreactivity (S1A Fig green box). Once the ROI was identified, the same ROI was applied to the consecutive stained sections in Visiopharm (S1C Fig). For analysis done with the MATLAB toolbox, a circular ROI was depicted from the central point (red cross) guided from the Visiopharm Iba-1-defined ROI (S1B and S1D Fig).

All microglial labelled sections were analysed (Iba-1, CD68 and MHCII) in addition to GFAP, PLP, Coll IV, fibrinogen, and AQP4-immunolabelled sections with the MATLAB toolbox. For Iba-1, CD68, MHCII, GFAP, PLP and Coll IV an automatic segmentation algorithm based on [18] was implemented. This generated binary images from the scanned brightfield colour images, i.e. a value of 1 was assigned to the pixels where the stain of interest was present and 0 where it was absent. The first step of the segmentations was to apply colour deconvolution to change the representation of the RGB images to DAB, haematoxylin, and background channels [19]. Only the DAB intensities were kept, leaving a single-channel image. The second step was smoothing the image, for which we applied a Gaussian filter (σ = 2 pixels) to the DAB channel. Finally, we applied a global threshold to the filtered DAB channel to get the segmented binary image [20]. The resultant data was represented as an Area Fraction of staining ranging from 0–1. Alternatively to segmentation, for fibrinogen and AQP4 staining the percentage of stain in the ROI was determined by computing the average intensity of the DAB channel of such images.

Additionally, using Visiopharm for each section labelled with microglial markers (Iba-1, CD68 and MHCII), 5 images at 20x magnification (each image area 0.925mm^2^) were sampled randomly throughout the WM ROI (total sampled area per section: 4.64mm^2^) (S2 Fig). All sampled images were fed into a Linear Bayesian detection algorithm within Visiopharm to determine the area of positive stain (labelled pixels). An average area fraction across all 5 images was generated for each case. Alternatively, for PLP, fibrinogen and AQP4 labelled sections the positively labelled structures (labelled pixels) were determined as a percentage of the total area of the ROI.

For all dual-labelled staining within the DSCL, 10 random fields were captured using a Nikon Eclipse 80i microscope (Nikon UK, Kingston Upon Thames). Images were transferred to a PowerPoint programme where a grid was overlaid on each image. The number of CD68^+^, Iba-1^+^ and CD68^+^/Iba^+^ colocalised cells were assessed, allowing for the percentage of microglia cells that were Iba-1^-^/CD68^+^ to be determined.

#### Semi-quantitative fibrinogen analysis

Fibrinogen staining patterns were also assessed semi quantitatively in the identified ROI by two independent observers (RW & JRH). This generated two scores: Firstly, the extent of parenchyma/perivascular staining (S3A Fig) was assessed as none (G0), perivascular staining only (G1), or immunoreactivity throughout the WM ROI, intense (I) and less intense (L) respectively (G2I or G2L). Secondly, the overall fibrinogen immunoreactivity (S3B Fig) was graded as follows; very faint overall immunoreactivity with the odd immunopositive glial cell, axonal/capillary tract with possible perivascular deposition of fibrinogen (G0). Overall more immunopositive axonal/ capillary tracts alongside possible perivascular deposition of fibrinogen with fewer immunopositive cells (G1). Overall more immunopositive cells with fewer immunopositive axonal/capillary tracts alongside possible perivascular deposition of fibrinogen (G2). Intense cell specific and axonal/capillary tract immunoreactivity throughout the ROI (G3).

### Microglial morphology metric

Microglial phentotype profiles were quantitatively assessed using a metric that considered both shape and size of the individual cells. We defined the ‘morphology (M) metric’ for quantifying microglial cell morphology along the ‘ramified’ to ‘amoeboid’ spectrum as follows,

$$M_{i}=\{\begin{array}{ll} C_{i}\frac{A_{i}-A_{0}}{A_{0}}, & if\;A_{i}\geq A_{0},\\ 0 & if\;A_{i}<A_{0}, \end{array}$$

where *i* indexes the cells in the image. *A*_*i*_ is the cell’s total area and *A*_*0*_ is the minimum area considered for the cells to be analysed. *c_i_* is the cell’s roundness which is defined as $c_{i}=\frac{4\pi A_{i}}{{P_{i}}^{2}}$, where *P*_*i*_ is the cell’s perimeter.

For each sample, we computed the mean score

$$\bar{M}=\frac{1}{N}\sum_{i=1}^{N}M_{i}$$

Where *N* is the total number of cells with *A* > *A*_*0*_ in a sample.

**Fig 1:**
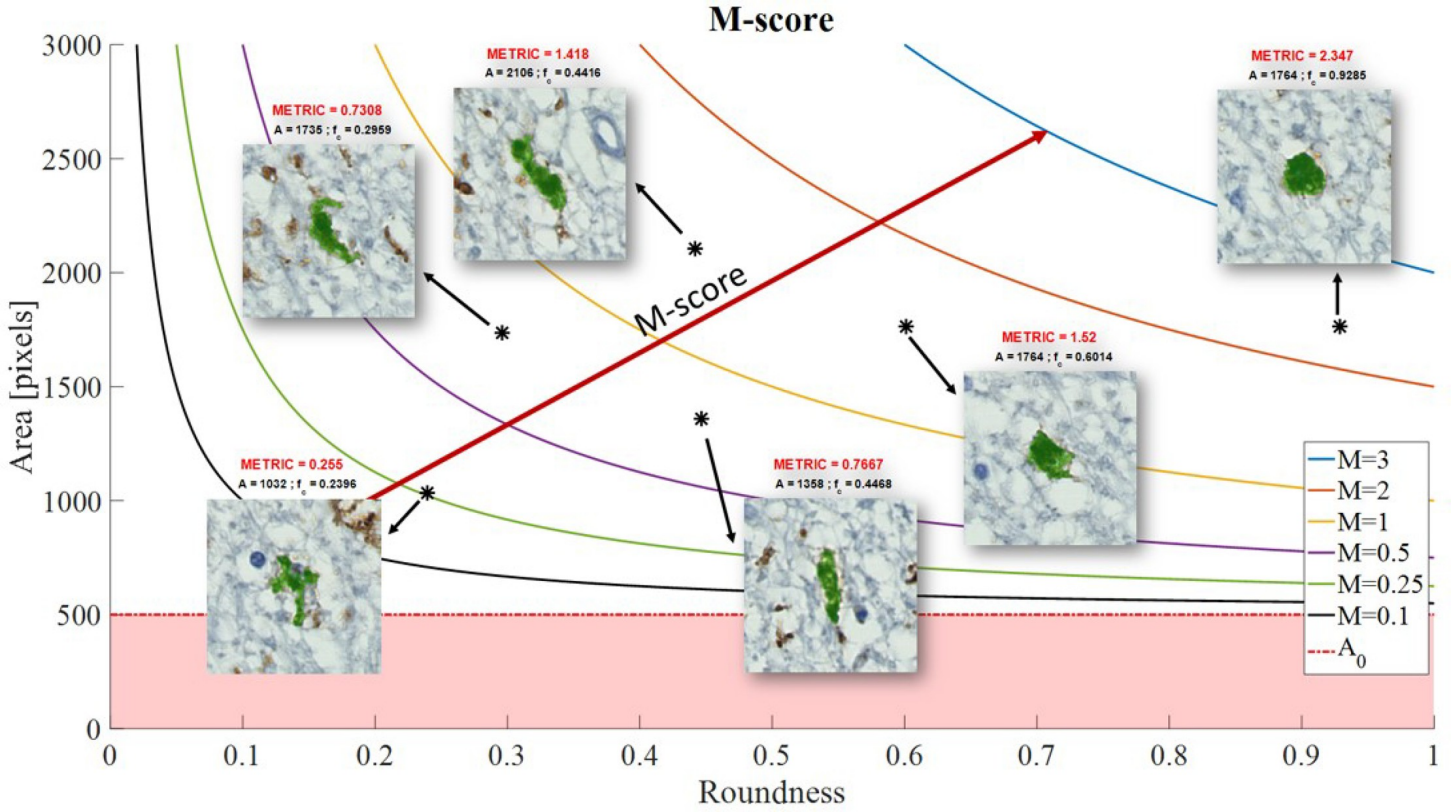
Microglial morphology metric. Each positively stained microglial was assigned a M-score, based on the area and roundness of the individual immunopositive cell. The less round and smaller the cell (more resting, ramified microglia) resulted in a smaller M-score. The more round and larger the cell (more amoeboid, phagocytic microglia) resulted in a larger M-score.

The M-score gives a higher value to more larger and rounder structures. An overall threshold area of 500 pixels was set for all three microglia markers Iba-1, CD68 and MHCII. Therefore, structures whose area was smaller than 500 pixels (26.45 μm^^2^) have a score equal to zero. Structures below this threshold were not specific to individual cells, picking out transverse microglial processes and therefore these were not considered in the overall analysis. As the structures/cells increase in size (area), so does the metric (Fig 1).

### Statistical analysis

Spearman’s rank correlation was used to assess the relationship between the separate histoscores from both the Visiopharm and MATLAB toolbox analyses of microglial density. The relationship between the different markers within the different patient groups was determined using a non-parametric Kruskal-Wallis test with the Dunn's multiple comparison test. For fibrinogen semi-quantitative analysis, interobserver variability was assessed using weighted kappa and differences between the WM groups were determined using Chi Square. All graphs and data analysis was completed in GraphPad Prism version 7 (GraphPad Prism In., USA).

## Results

### A significant difference in Iba-1 and CD68 immunoreactivity but not MHCII immunoreactivity is apparent across the three white matter groups

**Fig 2:**
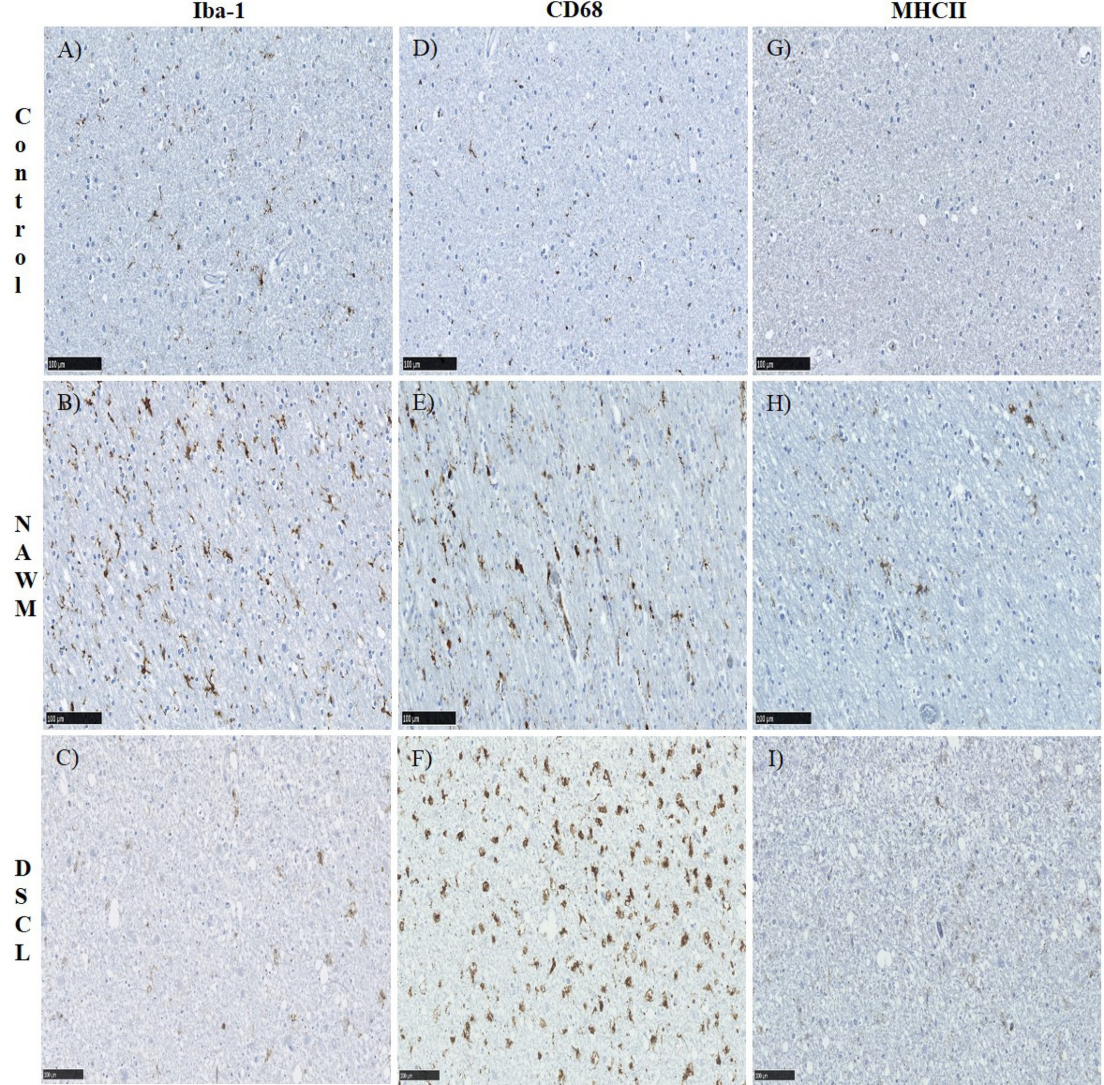
Microglial immunostaining. Iba-1 (A-C), CD68 (D-F) and MHCII (G-I) protein expression within the control, NAWM and DSCL cases. Control WM showed a regular distribution of Iba-1 positive ramified microglia (A) in contrast to the little CD68 and MHCII immunostaining in control WM (D & G). Within NAWM an increase in immunoreactivity was evident across all three microglial markers (B,E,H). Within DSCL, Iba-1 and MHCII immunostaining highlighted both ramified and amoeboid microglia (C & I). In contrast CD68 immunostaining highlighted amoeboid microglia (F). Scale bar = 100μm. NAWM: normal appearing white matter, DSCL: deep subcortical lesion, WM: white matter, MHCII: major histocompatibility complex II.

**Fig 3:**
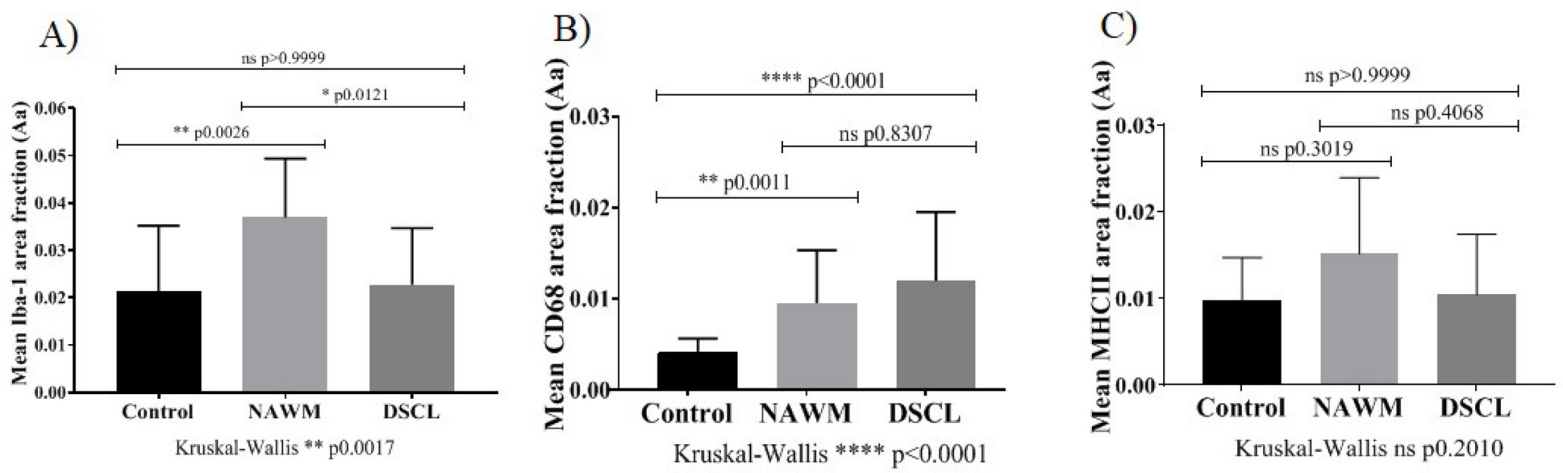
Quantification of microglial cell expression. The immunoreactive mean area of Iba-1 (A), CD68 (B) and MHCII (C) across the cohort. *P<0.05, **P<0.005 ***<0.001 ****P<0.0001. Error bars indicate standard deviation (SD) of the mean. MHCII: major histocompatibility complex II, ns: non-significant.

The immunoreactive profile of Iba-1, CD68 and MHCII was assessed across the three groups. In non-lesional control cases, Iba-1 immunolabelled ramified, bipolar cells throughout the WM (Fig 2A). This pattern of Iba-1 immunoreactivity was also a feature of NAWM, in addition to larger cells with retracted cell processes, consistent with the morphology of “primed microglia” (Fig 2B). Within DSCL a combination of both Iba-1 immunopositive, ramified and larger amoeboid, round cells were present (Fig 2C). In comparison, there were very few CD68^+^ cells in control cases (Fig 2D) with an increase in CD68 immunoreactivity in the NAWM cases presenting immunopositive cells with retracting processes (Fig 2E), through to the enlarged, swollen, amoeboid cells present in the DSCL (Fig 2F). CD68 immunoreactivity within DSCL was prominent in the cell body indicative of a phagocytic microglial phenotype. MHCII immunoreactivity was scarce in control cases (Fig 2G), with a slight increase in immunoreactivity of the typical ramified microglial cell profile within NAWM cases (Fig 2H) with very little difference in immunoreactivity seen between the NAWM and DSCL cases (Fig 2I). However, a few MHCII^+^ amoeboid cells were present amongst the limited number of ramified MHCII^+^ cells in the DSCL. For quantitative analysis of each of the different microglial markers, we compared two different analysis packages; our own system within the MATLAB toolbox and a Visiopharm application. The packages strongly correlated with each other when assessing the microglial immunoreactivity (Spearman’s rank CD68 r = 0.920, p = 0.0001; Iba-1 r = 0.926, p = 0.0001; MHCII r = 0.801, p = 0.0001). Henceforth, all results presented in this paper are from the MATLAB toolbox unless otherwise stated. Both Iba-1 and CD68 staining differed between control, NAWM and DSCL groups (Fig 3A and 3B) respectively (Kruskal-Wallis Iba-1; p = 0.0017, CD68; p = 0.0001). However, no difference was seen in MHCII staining across the three groups (Fig 3C) (Kruskal-Wallis MHCII; p = 0.2010). Quantification of Iba-1 immunoreactivity showed significantly higher levels of Iba-1 immunoreactivity in NAWM vs. controls (p = 0.0026) and a significantly reduced expression in DSCL vs. NAWM (p = 0.0121), Fig 3A. A significant increase in CD68 immunoreactivity was seen in NAWM vs. controls (p = 0.0011) and in DSCL vs. controls (p<0.0001) with no significant difference in expression in DSCL vs. NAWM, Fig 3B. When comparing both Iba-1 and CD68 immunoreactivity it is worth noting that the overall total immunoreactive area of CD68 within DSCL regions was around half of that of Iba-1 within DSCL regions (S4 Fig). In summary, the Iba-1 microglial load was significantly increased in the NAWM group compared to controls and significantly decreased in the DSCL group compared to NAWM, to levels comparable to that seen in controls. In contrast, the CD68 microglial load was significantly increased in both the NAWM and DSCL groups compared to controls. No significant difference was identified in the expression of MHCII across the groups (Fig 3C).

### The morphology of Iba-1^+^ and CD68^+^ but not MHCII^+^ microglia significantly varies between the three white matter groups

**Fig 4:**
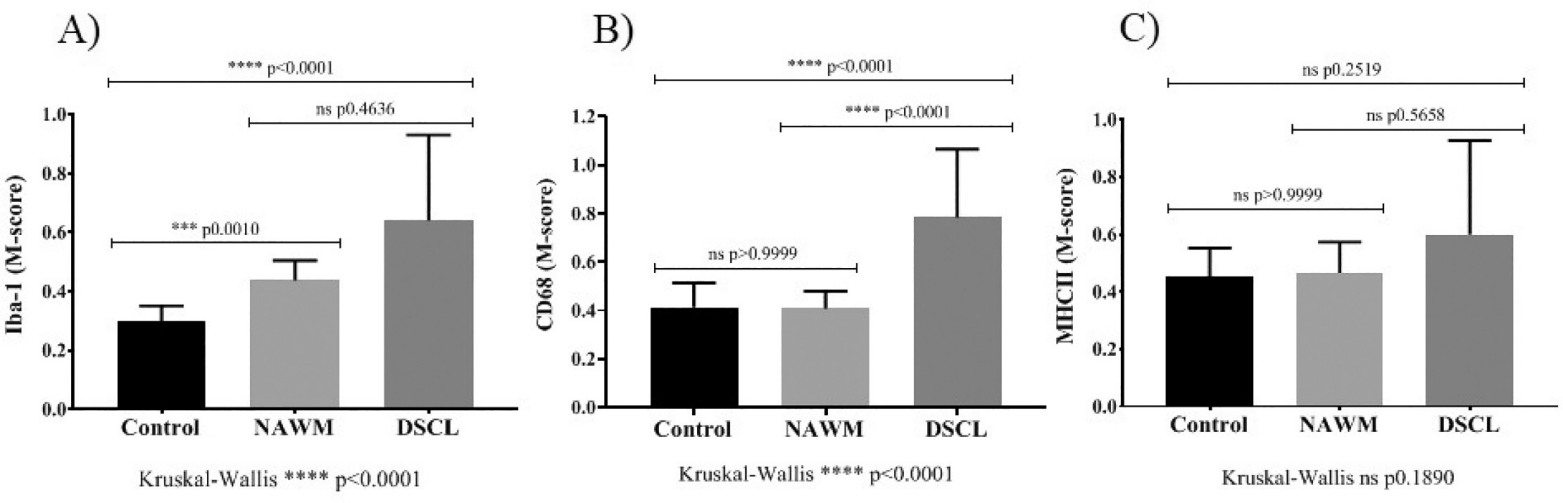
Microglial morphology analysis. Iba-1 labelled microglial increase in size and roundness (M-score) throughout the three groups, indicative of an increasing amoeboid, phagocytic phenotype between DSCL vs NAWM and NAWM vs control cases (A). CD68-labelled microglia also increase in size and roundness, indicative of amoeboid, phagocytic phenotype between DSCL vs NAWM and control cases (B). MHCII-labelled microglia show no significant difference in their M-score between the three groups (C). *P<0.05, **P<0.005 ***<0.001 ****P<0.0001. Error bars indicate standard deviation (SD) of the mean. NAWM: normal appearing white matter, DSCL: deep subcortical lesion, MHCII: major histocompatibility complex II, ns: non-significant.

In each ROI, the shape of microglia along the ‘ramified-amoeboid’ axis was assessed using the morphology metric (‘M-score’) for each microglial marker (refer to Fig 1). The shape of Iba-1-labelled microglia differed between the three groups (Kruskal-Wallis Iba-1; p = 0.0001) with a significantly increased Iba-1 microglia M-score in NAWM vs. control (p = 0.0010) and DSCL vs. control (p = 0.0001), while no significant difference in the M-score was seen between DSCL vs. NAWM (Fig 4A).

The shape of CD68^+^ microglia differed between the three groups (Kruskal-Wallis CD68; p = 0.0001) with a significantly increased CD68 microglia M-score in DSCL vs. NAWM (p = 0.0001) and DSCL vs. control (p = 0.0001). No difference in CD68 microglial M-score was observed between the NAWM vs. control cases (Fig 4B). In contrast, there was no significant difference identified in the M-score of MHCII^+^ microglia between the three groups (Kruskal-Wallis MHCII; p = 0.1890) (Fig 4C).

In summary, the M-score showed significant changes along the ramified to amoeboid axis from control to NAWM and control to DSCL for Iba-1^+^ microglia. For CD68^+^ microglia, there was an increase in M-Score from control to DSCL and NAWM to DSCL regions, but no significant increase from control to NAWM. No difference in the M-score was identified for MHCII^+^ microglia.

### Not all CD68^+^ microglia express Iba-1

**Fig 5:**
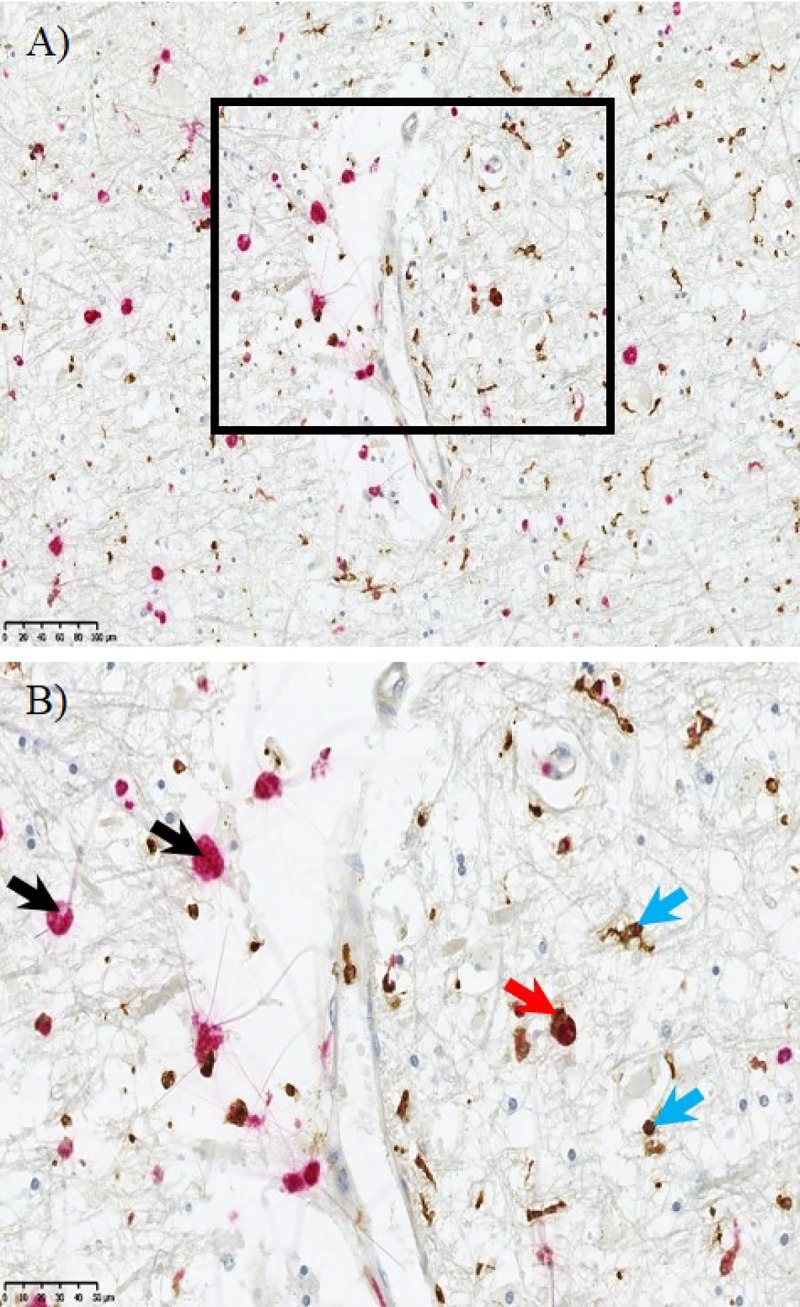
Microglial immunohistochemistry. Distinct microglial cell populations are evident within the deep subcortical lesion (DSCL). CD68^+^ amoeboid microglial (red) were indicated by the black arrow and separate more ramified cells were labelled with Iba-1 (brown) indicated by the blue arrow. Dual staining showed some colocalisation of CD68 (red) and Iba-1 (brown) indicated by the red arrow. Scale bar = 50μm.

As noted earlier, there appeared to be reduced Iba-1^+^ but increased CD68^+^ area fraction in DSCL compared to NAWM. This seemed counter intuitive given the suggestion that Iba-1 is a pan microglial marker. To resolve this, double-labelling IHC was performed. This revealed that the majority of the large, swollen amoeboid microglia were CD68 immunopositive. While a number of these CD68 immunopositive cells were also Iba-1^+^ (Fig 5, red arrow) but others were Iba-1^-^ (Fig 5, black arrows). This was also confirmed using immunofluorescence (S5 Fig). In a representative case following quantification, approximately 32.5% of the total microglia in DSCL were CD68^+^/Iba-1^-^. Alongside the amoeboid microglia within the DSCL were the typical ramified microglia, the majority of which were Iba-1 immunopositive (Fig 5, blue arrows). Thus it appears that in the transition from NAWM to DSCL, there is an increase in the numbers of microglia with CD68 expression with a corollary shift from ramified to amoeboid morphology and a contrasting loss of Iba-1 expression.

### Myelin integrity is reduced in DSCL compared to control and NAWM

**Fig 6:**
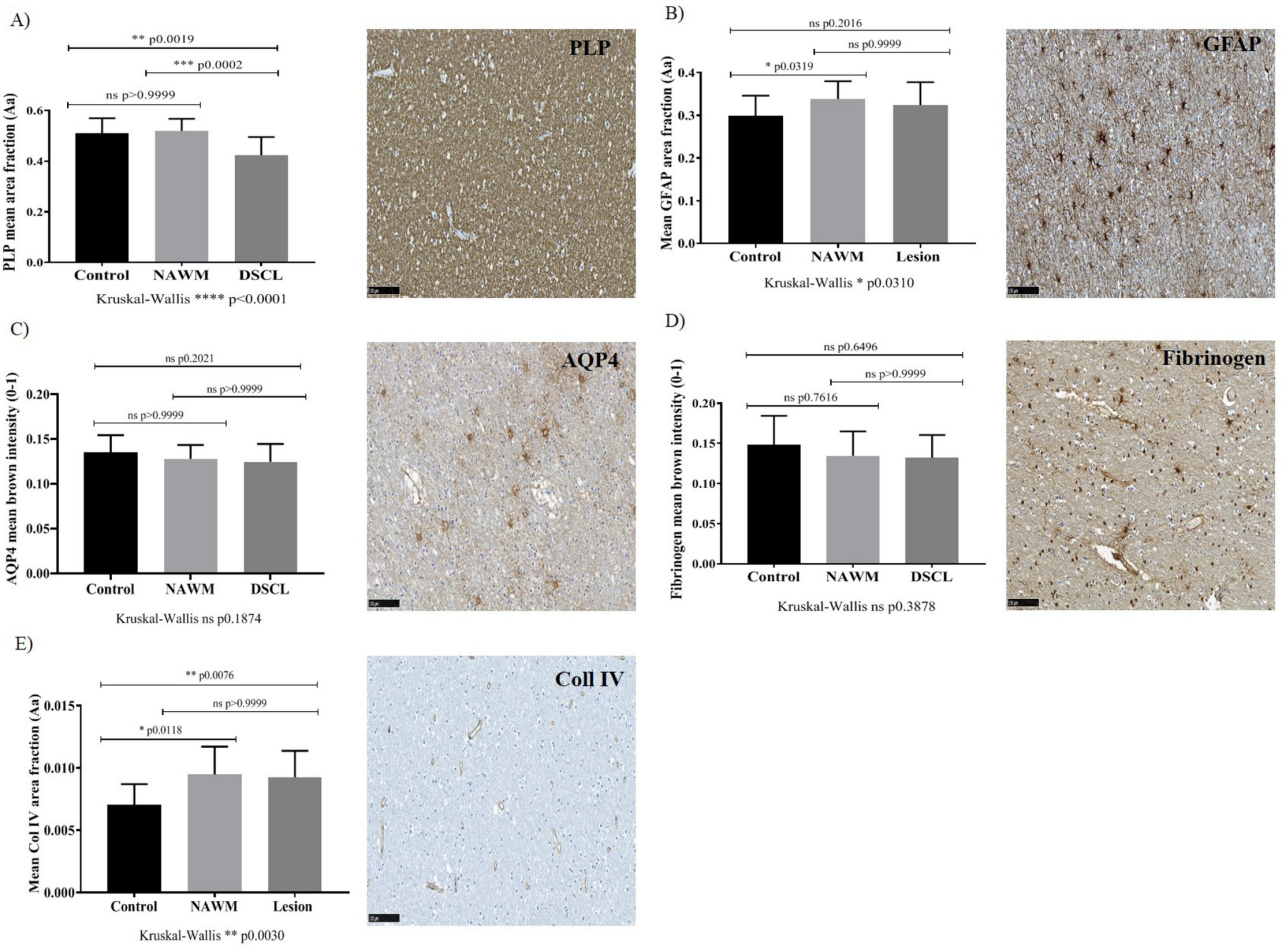
A loss of vascular integrity does not contribute to WM pathology. A decrease in PLP expression in DSCL vs control and DSCL vs NAWM (A).GFAP immunoreactivity increases in NAWM vs controls (B). No significant differences in AQP4 (C) and fibrinogen (D) are apparent across the cohort. A subtle increase in Coll IV in NAWM vs control and DSCL vs control was identified (E). *P<0.05, **P<0.005 ***<0.001 ****P<0.0001. Error bars indicate standard deviation (SD) of the mean. PLP: proteolipid protein, DSCL: deep subcortical lesion, NAWM: normal appearing white matter, GFAP: glial fibrillary acidic protein, AQP4: aquaporin 4, Coll IV: collagen IV, ns: non-significant.

Myelin integrity was assessed by PLP immunostaining which differed significantly between the control, NAWM and DSCL groups when analysed (Kruskal-Wallis PLP; p<0.0001). PLP immunostaining was significantly lower in DSCL vs. NAWM (p = 0.0002) and in DSCL vs. controls (p = 0.0019) (Fig 6A). Both analysis packages (MATLAB toolbox and Visiopharm application) strongly correlated with each other when assessing the extent of PLP immunoreactivity, Spearman r = 0.8395, p<0.0001. Additionally the expression of PLP negatively correlated with CD68 expression (r = -0.3117, p = 0.0210).

### A loss of vascular integrity does not contribute to microglial changes in the brain

Given that the expression of the microglia markers Iba-1 and CD68 differ across the lesion cohort and PLP expression negatively correlates with CD68 immunoreactivity (as PLP expression decreases the expression of CD68^+^ microglia increases) it was important to investigate the potential causes of these changes. Recently it has been suggested changes in vascular integrity within the brain may cause demyelination and the associated changes in glia that maybe contributing to or preventing DSCL formation. Consequently, we investigated the expression of a range of vascular markers in our patient cohort including: Coll IV, a marker of vessel basement membrane; fibrinogen, a serum protein used as a marker of vascular leakiness; and AQP4 a protein expressed on astrocyte end feet that regulates the transport of water and is believed to contribute to vascular integrity in the brain. GFAP immunoreactivity was significantly higher in NAWM vs. control cases (p = 0.0319) (Fig 6B) and correlated with the three microglial markers across the whole cohort (GFAP vs. Iba-1 r = 0.2644, p = 0.0530, GFAP vs. CD68 r = 0.3208, p = 0.0180, GFAP vs. MHCII r = 0.354, p = 0.0136), AQP4 expression did not appear to be significantly altered across the groups (Fig 6C). Additionally, we found no significant difference in the area density of fibrinogen deposition across the cases investigated (Fig 6D). Similarly, the analyses of the pattern and intensity of fibrinogen deposition did not differ significantly between groups when rated by either observer (all P ≥ 0.067 by Chi square): Fibrinogen immunoreactivity was present and varied across the whole cohort (S3 Fig) suggesting serum protein deposition in the ageing brain regardless of WML status. However, there was a difference in Coll IV immunoreactivity across the patient cohort (Kruskal-Wallis; p = 0.0030): There was greater Coll IV immunoreactivity in NAWM vs. control (p = 0.0180) and DSCL vs. control cases (p = 0.0076) (Fig 6E). In summary, while there is initial evidence for vascular basement membrane changes, evidence of a loss in vascular integrity contributing to changes in microglial pathology are not apparent.

## Discussion

Microglia are diverse cells that express a range of different markers associated with their particular function and role within the CNS [9]. This current study uses two separate image analysis platforms to quantitate Iba-1, CD68 and MHCII immunoreactivity across control, NAWM and DSCL of an ageing cohort. While NAWM has greater levels of Iba-1 microglial immunoreactivity than control brains, the formation of a DSCL against a background of NAWM appears to be associated with a loss of Iba-1 immunoreactivity in microglia. This conflicts with the recurring acceptance in the literature of Iba-1 as a pan microglial marker [13]. On the other hand, an expected increase in CD68 immunoreactivity was identified with progression from control, to NAWM to DSCL.

With specific cellular staining associated with all three microglial markers the image analysis carried out by both the MATLAB toolbox and Visiopharm application were highly comparable and generated similar intergroup differences.

Hopperton *et al* recently reviewed the literature surrounding microglia markers in human post-mortem brain samples from control subjects and patients with Alzheimer’s disease (AD), which is known to be associated with WML [21]. The review concluded that of the existing studies, 76/113 measured microglia using one of the three common markers; Iba-1, CD68, and MHCII. Consistent upregulation of CD68 and MHCII was identified in AD compared to control brains. This supports our finding of increased CD68 expression in NAWM and DSCL regions but contrasts with our finding of no differences of MHCII expression across the three WM groups. None the less 7/43 studies in the Hopperton *et al* review also showed no difference in MHCII expression between AD and control across multiple brain regions. It should be noted that the population-based design of CFAS contrasts with the Hopperton *et al* meta-analysis, which drew studies that focussed on AD. Thus raising the possibility that the relationship of MHCII with WML may vary as a function of Alzheimer pathology. Of the 20 studies that compared Iba-1, 10 studies showed no difference or a decrease in Iba-1 in AD relative to control brains coinciding with our finding of relatively similar levels of Iba-1 in the DSCL of an ageing cohort compared to control cases. It would thus appear that a loss of microglial Iba-1 is associated with pathological, neurodegenerative states.

Iba-1, a cytoplasmic protein that reflects microglial motility and migration, is widely regarded as a pan-microglial marker [13, 14, 22]. In the current study, the total Iba-1 immunoreactive area increases in NAWM regions surrounding DSCLs compared to control regions, but decreases in the transition from NAWM to DSCL. This may imply increased migration of microglia as a ‘field change’ in NAWM from brains with DSCL, followed by a loss of motility and migration that correlates with DSCL formation.

Whether these Iba-1^-^ microglia are adopting a neurotoxic or neuroprotective phenotype is unknown. Future work to determine the M1/M2 phenotype of these cells is required. However, it is known that as the microglia adapt a phagocytic phenotype, they increase in size and roundness. This has been documented in NAWM where subtle pathological changes occur including gliosis, myelin and axonal changes etc. [12, 23, 24]. Thus, it is interesting that our morphology index which assesses size and shape (with higher scores corresponding to larger, less ramified, more rounded forms) of the Iba-1^+^ microglia shows stepwise increases throughout the tissue groups. This, together with the increasing CD68 immunoreactivity suggests the increasing phagocytic phenotype of microglia from control, NAWM through to DSCL. Our histological results show that Iba-1 immunolabels two distinct populations of microglia: those with a ramified profile present in high numbers in the NAWM and as well as phagocytic microglia, a prominent feature of the DSCL and that are a very prominent feature of CD68^+^ microglia.

We recognise that cell morphology alone only provides a partial insight into microglial function and that assessment of the expression of additional microglial markers is crucial to fully characterise microglial phenotype [10]. However, we maintain, on the basis of our results, that combining the morphological status of the cells with assessment of marker expression improves understanding of the microglial function.

The increase in the size and roundness of CD68^+^ cells from NAWM to DSCL regions is an additional indication of their phagocytic phenotype. This finding replicates previous studies that have demonstrated amoeboid CD68^+^ microglia as a prominent feature in DSCL [7]. Whether the amoeboid phagocytic microglia are causing harm by actively phagocytosing functioning cells, or clearing up debris from damaged axons and degraded myelin therefore responding to neurodegeneration is open to question [10].

When comparing both Iba-1 and CD68 immunoreactivity it is worth noting that the overall total immunoreactive area of CD68 within DSCL regions was around half of that of Iba-1. Therefore, the question rises as to what microglial populations account for the overall Iba-1^+^/CD68^-^ immunoreactive area. Comparison of the shape metrics of the Iba-1 and CD68 immunopositive cells, indicates there are greater numbers of CD68^+^ cells with the larger/less ramified morphology than is seen for Iba-1^+^ microglia. Consequently, the remaining Iba-1^+^/CD68^-^ cells within the DSCL must be of the more ramified, bipolar phenotype. These differences in CD68 and Iba-1 shape metrics further support our finding that not all CD68^+^ cells are Iba-1^+^, despite the general consensus that Iba-1 is a pan-marker of microglia.

Whether Iba-1 expression loss by CD68^+^ cells is a feature that is specific to DSCL in ageing or a phenomenon of WML in general is currently unknown. However, this hypothesis has recently been supported by work looking at the relationship between microglial immunophenotype in dementia in the context of Alzheimer’s pathology [10]. This study concluded that the presence of dementia correlated positively with CD68 levels but negatively with Iba-1 levels, and that different microglial populations may coexist within the brain. Whether the microglia in the DSCL are a new type of cell that are Iba^-^/CD68^+^ arising as a result of the lesion formation or whether the microglia lose their Iba-1 expression as a result of lesion formation requires additional testing and examination in other disease processes in a quantitative manner.

The association of WM pathology with BBB dysfunction has been assessed previously through the detection of serum proteins including fibrinogen, IgG and albumin outside the vasculature in the brain parenchyma [3, 25]. In the current study, large inter individual differences in extravascular fibrinogen expression were apparent and there was clear evidence for serum accumulation in the ageing WM, yet there was no relationship between these index of BBB dysfunction and WM pathology. The literature to date on this matter is conflicting–some studies find evidence for serum protein accumulation in the brain, while others do not [26–28].

Our work showed no apparent differences in AQP4 expression across the cases. This finding suggests no evident change in astrocytic water transport out of the interstitium. This finding is in line with our lack of evidence of vascular leakiness as indexed by fibrinogen IHC described above. Chen *et al* have demonstrated that there is loss of AQP4 in astrocytic end-feet in WML of post stroke patients with dementia compared to the WML of post stroke patients without dementia [29]. Therefore, future studies could focus on whether AQP4 expression differentiates between demented and non-demented patients with DSCL. However, we lack the sample size and case numbers to test this hypothesis here.

We have demonstrated increased levels of Coll IV immunoreactivity in both NAWM and DSCL compared to control regions. An increase in Coll IV could be due to a number of factors, namely an increase in basement membrane thickness or an increase in the number of vessels. Increased basement membrane thickness has been suggested to serve as a protective measure to prevent vascular leakiness and consequently fibrinogen and other serum protein deposition and accumulation [30]. Furthermore, an increase in vessel thickness may impede cerebral blood flow contributing to WML development [31]. Secondly, the increase in Coll IV could be due to the increased number of vessels in the NAWM and DSCL over the controls as a result of pathology. Alternatively, the demyelinating lesions could result in ‘compacting’, reducing intervessel distance and subsequently increasing Coll IV immunoreactivity within the DSCL. Evidence has shown the tortuosity (lengthening) of arterioles in the deep WM in areas where brain parenchyma has been lost, particularly in the aged brain [32, 33]. The nature of the Coll IV alterations, including the possibilities of greater basement membrane thickness or numbers of blood vessels is currently being investigated by our laboratory.

## Conclusions

In summary, this study identifies that some, but not all, microglia markers correlate with the progression from control WM in the ageing brain to NAWM to fully-developed DSCL. Clearly when using IHC to investigate the proposed function of particular cell types in pathology it is important to look at a range of cellular markers. Initially, with progression from control to NAWM, there is an increase in both CD68^+^ and Iba-1^+^ microglia. Following this, with the step from NAWM to DSCL, there is a further increase in the CD68^+^ signal. However, uniquely the study also shows a distinct population of microglia within DSCL that lose their Iba-1 expression. This finding disputes the existing concept that Iba-1 is a pan-microglial marker. Identifying sub populations of microglia that differ based on the markers they express may ultimately help us to decipher their overall function and explain the underlying pathology of neurological diseases. Therefore, it is important this discovery is explored beyond that of the DSCL in the ageing brain to investigate whether this finding is apparent in all WML and other neurological diseases.

## Supporting information

S1 Fig Visiopharm vs MATLAB analysis. The ROI in each case was identified based on the extent of microglial activation (Iba-1 reactivity and/or CD68 reactivity). The ROI identified in a NAWM case using Iba-1 immunoreactivity in visiopharm (A). The circular ROI was depicted in the equivalent MATLAB processed image. The ROI identified from the central point (red cross) guided by the visiopharm Iba-1 defined ROI (B). The equivalent ROI represented on the fibrinogen labelled section for the same NAWM case in visiopharm (C). The circular ROI in the equivalent MATLAB processed fibrinogen image. The ROI was identified from the central point (red cross) guided from the visiopharm fibrinogen defined ROI (D). ROI: region of interest, NAWM: normal appearing white matter.
(TIF)

S2 Fig Visiopharm analysis. An example image uploaded into visiopharm (A) and the main region of interest (ROI) drawn onto the section (B, green box). The overall mean area of immunostaining was calculated across the 5 sub ROIs within the main ROI (C).
(TIF)

S3 Fig Semi-quantitative analysis of fibrinogen. The parenchyma fibrinogen immunoreactivity was graded as G0; absent parenchyma immunoreactivity, G1; perivascular immunoreactivity only, G2L or G2I (Less intense and Intense immunoreactivity); throughout the all parenchyma. Scale bar = 1mm (A). The pattern of fibrinogen immunoreactivity in the region of interest (ROI) was graded as G0; very faint immunoreactivity throughout the ROI, G1; more axonal/capillary immunoreactivity throughout the ROI (black arrows), G2; more cell specific immunoreactivity throughout the ROI (red arrows), G3; Intense axonal/capillary (black arrows) and cell specific staining (red arrows) throughout the ROI (B). Parenchyma staining showed fair agreement between scorers (κ = 0.31) while the overall white matter staining showed moderate agreement between scorers (κ = 0.59) Scale bar = 100μm.
(TIF)

S4 Fig Comparison of CD68 and Iba-1 immunoreactivity. The extent of Iba-1 and CD68 immunoreactivity across areas of control, NAWM and DSCL (A). Iba-1 and CD68 microglia M-score across areas of control, NAWM and DSCL, the higher the M-score the more larger and rounder the cell (B). Error bars indicate standard deviation (SD) of the mean. NAWM: normal appearing white matter, DSCL: deep subcortical lesion.
(TIF)

S5 Fig Microglial double labelling. CD68+ (A, red fluorescent label) and Iba-1 detected with 3,3’-diaminobenzidine (DAB) visualised under light microscopy (B, brown). Double labelling confirms colocalisation of CD68 and Iba-1 double-labelled cells (C, green arrows) but also shows a distinct population of Iba-1- cells (B, black arrows) that are CD68+ (C, white arrows) confirming not all CD68+ cells are Iba-1+. Scale bar = 50μm.
(TIF)

S1 Table Minimal data set. Visiopharm and MATLAB generated data (MHCII, CD68, Iba-1, AQP4, Fibrinogen and PLP).
(XLSX)
