# Supplementary figures and images for: Iba-1-/CD68+ microglia are a prominent feature of age-associated deep subcortical white matter lesions
Source: PLoS One. 2019 Jan 25;14(1):e0210888. doi: 10.1371/journal.pone.0210888 (PMC6347230; doi:10.1371/journal.pone.0210888)

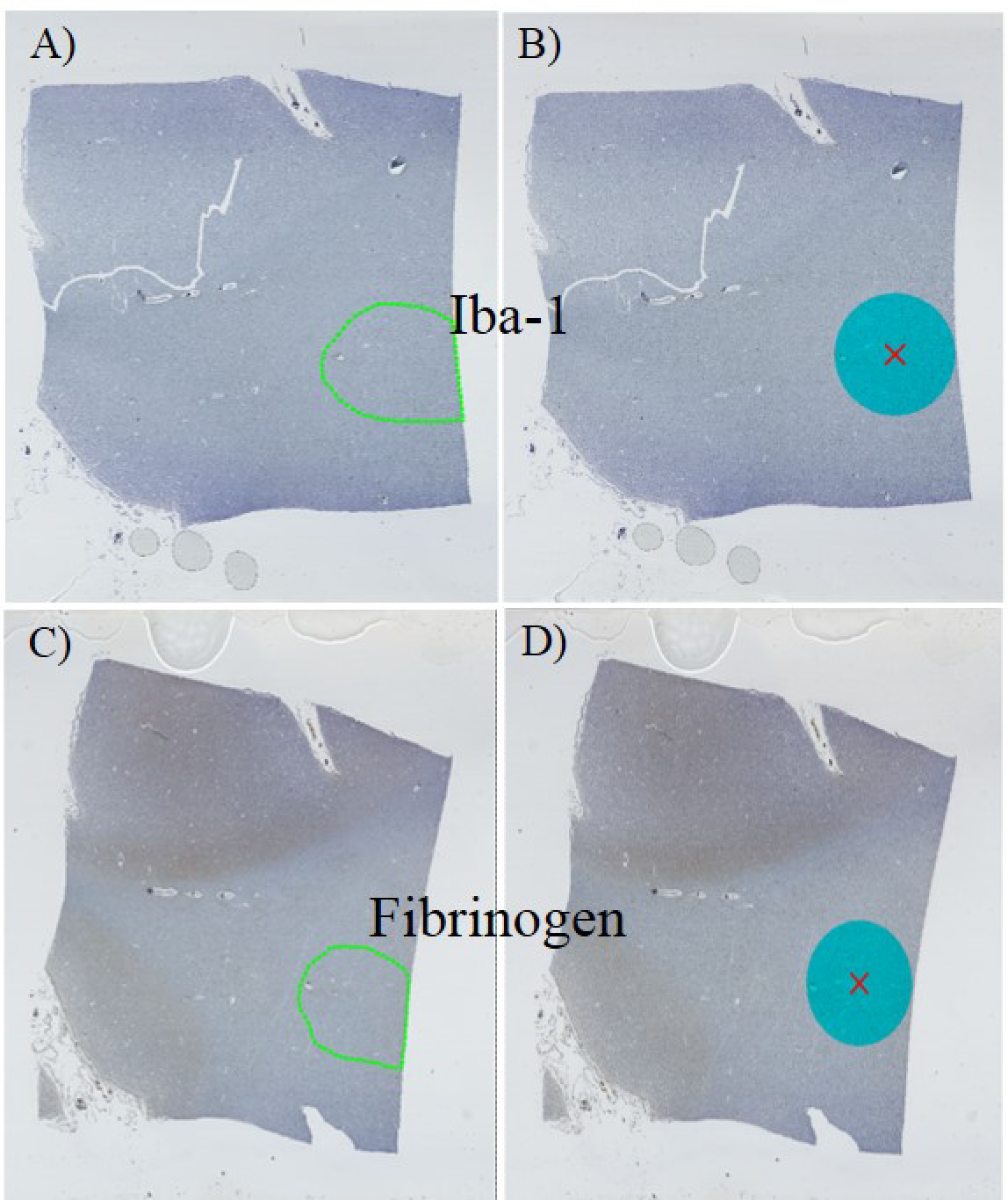

Supplement: S1 Fig — The ROI in each case was identified based on the extent of microglial activation (Iba-1 reactivity and/or CD68 reactivity). The ROI identified in a NAWM case using Iba-1 immunoreactivity in visiopharm (A). The circular ROI was depicted in the equivalent MATLAB processed image. The ROI identified from the central point (red cross) guided by the visiopharm Iba-1 defined ROI (B). The equivalent ROI represented on the fibrinogen labelled section for the same NAWM case in visiopharm (C). The circular ROI in the equivalent MATLAB processed fibrinogen image. The ROI was identified from the central point (red cross) guided from the visiopharm fibrinogen defined ROI (D). ROI: region of interest, NAWM: normal appearing white matter. (TIF) [file pone.0210888.s001.tif]

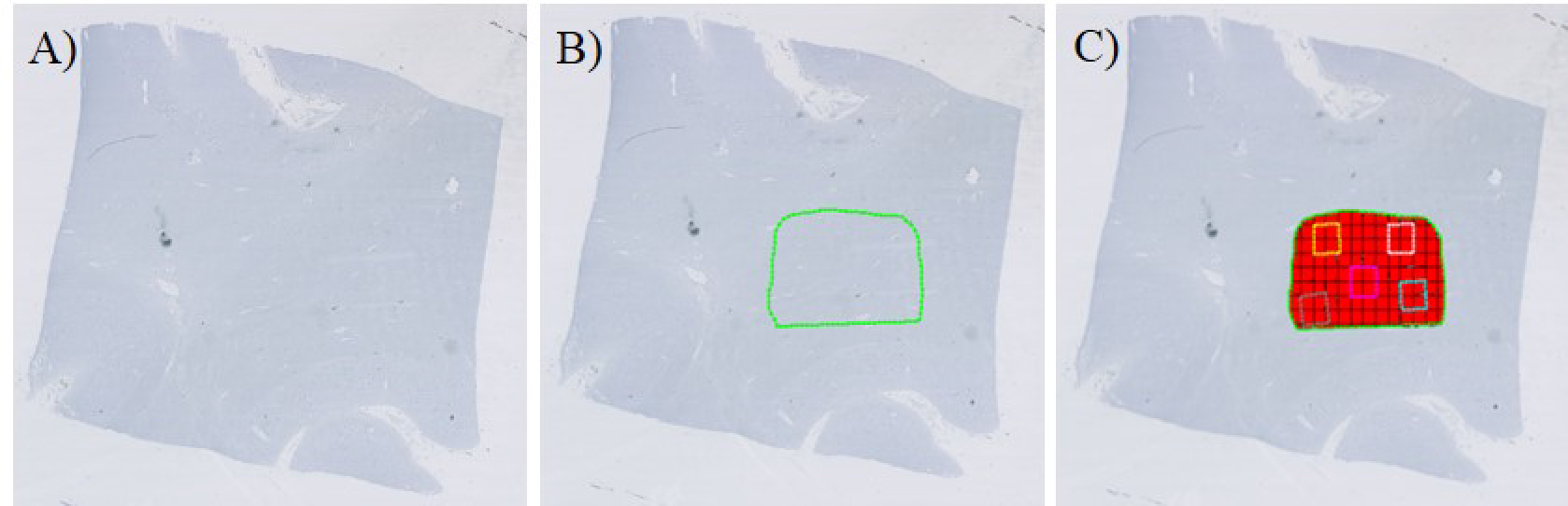

Supplement: S2 Fig — An example image uploaded into visiopharm (A) and the main region of interest (ROI) drawn onto the section (B, green box). The overall mean area of immunostaining was calculated across the 5 sub ROIs within the main ROI (C). (TIF) [file pone.0210888.s002.tif]

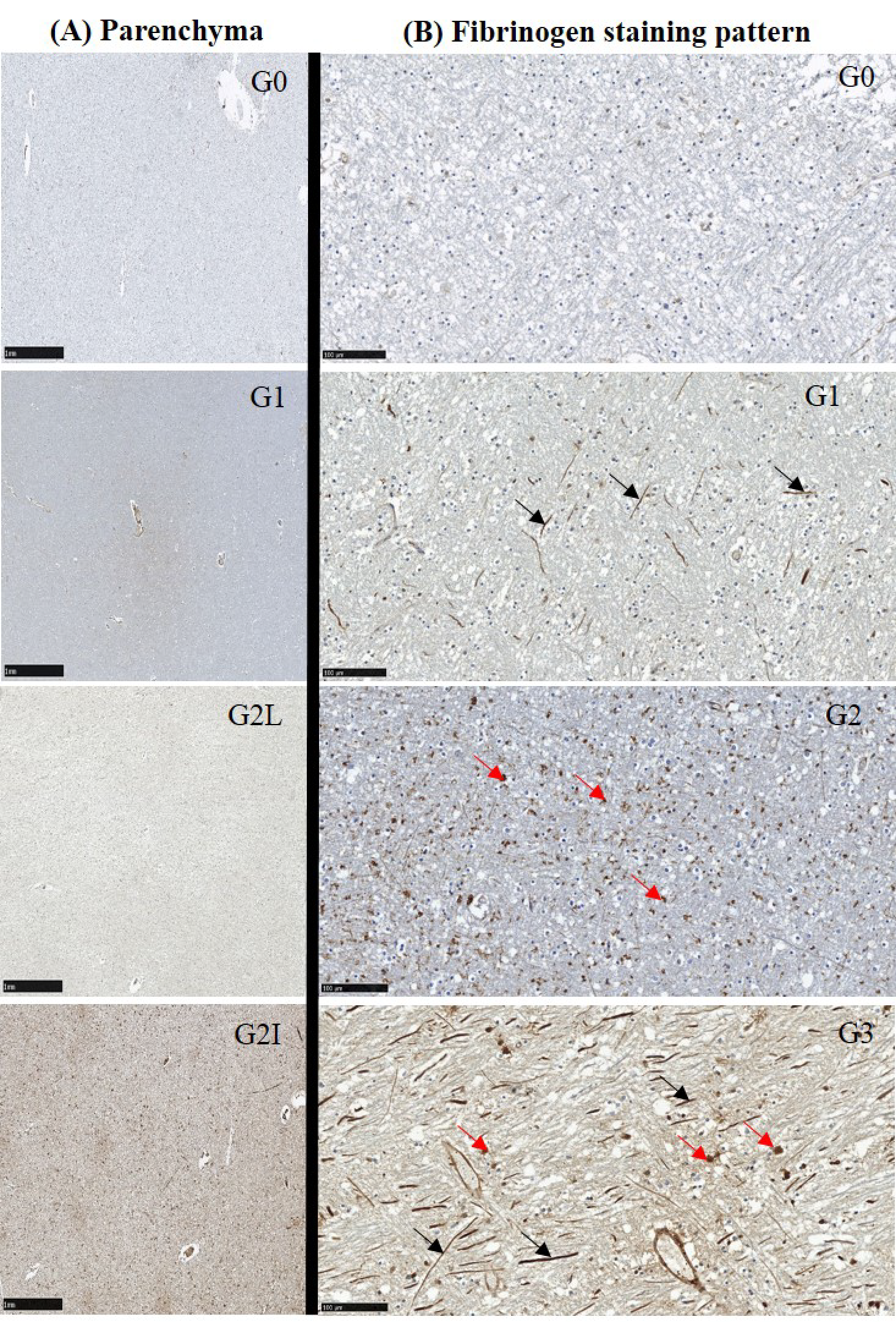

Supplement: S3 Fig — The parenchyma fibrinogen immunoreactivity was graded as G0; absent parenchyma immunoreactivity, G1; perivascular immunoreactivity only, G2L or G2I (Less intense and Intense immunoreactivity); throughout the all parenchyma. Scale bar = 1mm (A). The pattern of fibrinogen immunoreactivity in the region of interest (ROI) was graded as G0; very faint immunoreactivity throughout the ROI, G1; more axonal/capillary immunoreactivity throughout the ROI (black arrows), G2; more cell specific immunoreactivity throughout the ROI (red arrows), G3; Intense axonal/capillary (black arrows) and cell specific staining (red arrows) throughout the ROI (B). Parenchyma staining showed fair agreement between scorers (κ = 0.31) while the overall white matter staining showed moderate agreement between scorers (κ = 0.59) Scale bar = 100μm. (TIF) [file pone.0210888.s003.tif]

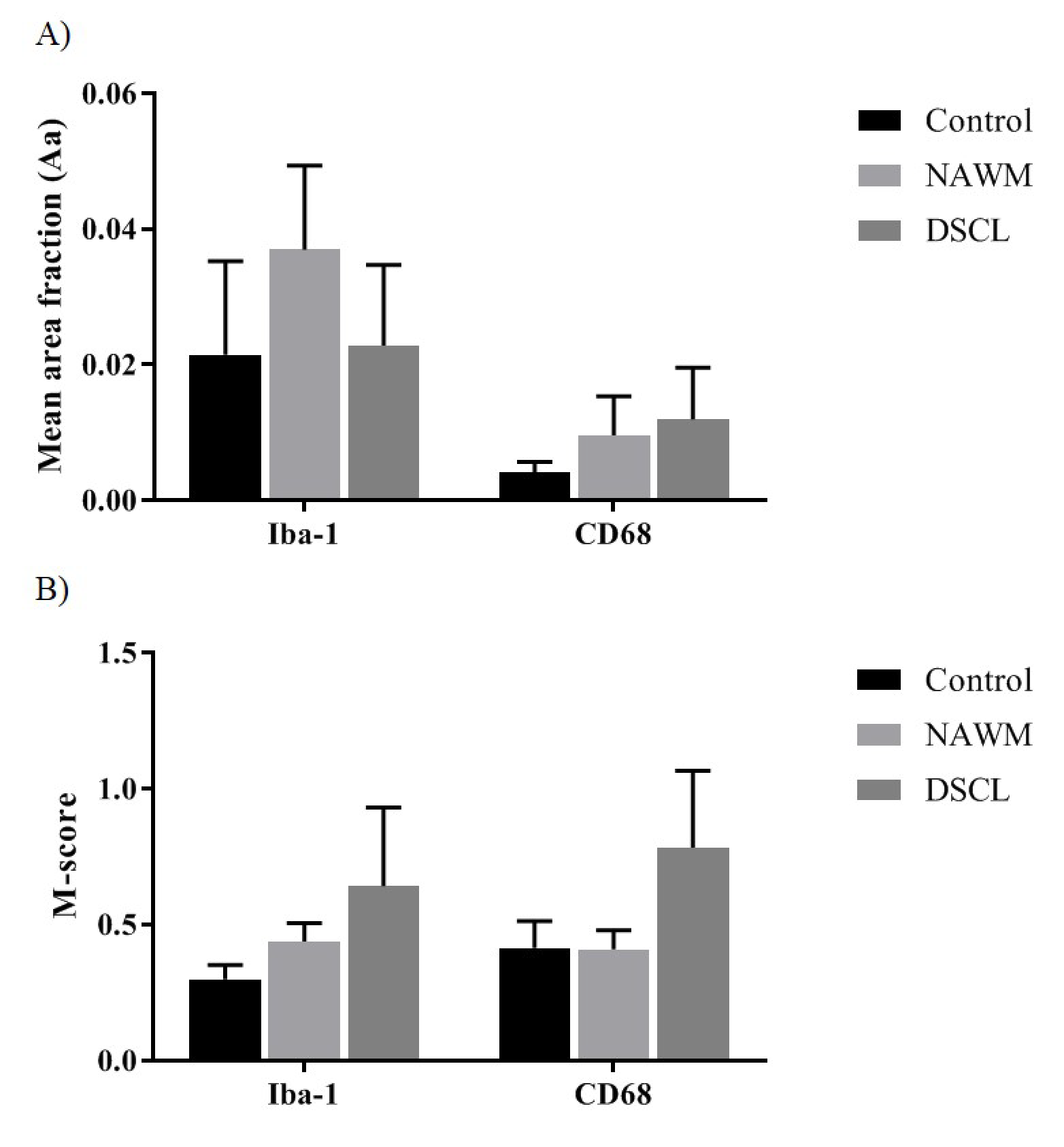

Supplement: S4 Fig — The extent of Iba-1 and CD68 immunoreactivity across areas of control, NAWM and DSCL (A). Iba-1 and CD68 microglia M-score across areas of control, NAWM and DSCL, the higher the M-score the more larger and rounder the cell (B). Error bars indicate standard deviation (SD) of the mean. NAWM: normal appearing white matter, DSCL: deep subcortical lesion. (TIF) [file pone.0210888.s004.tif]

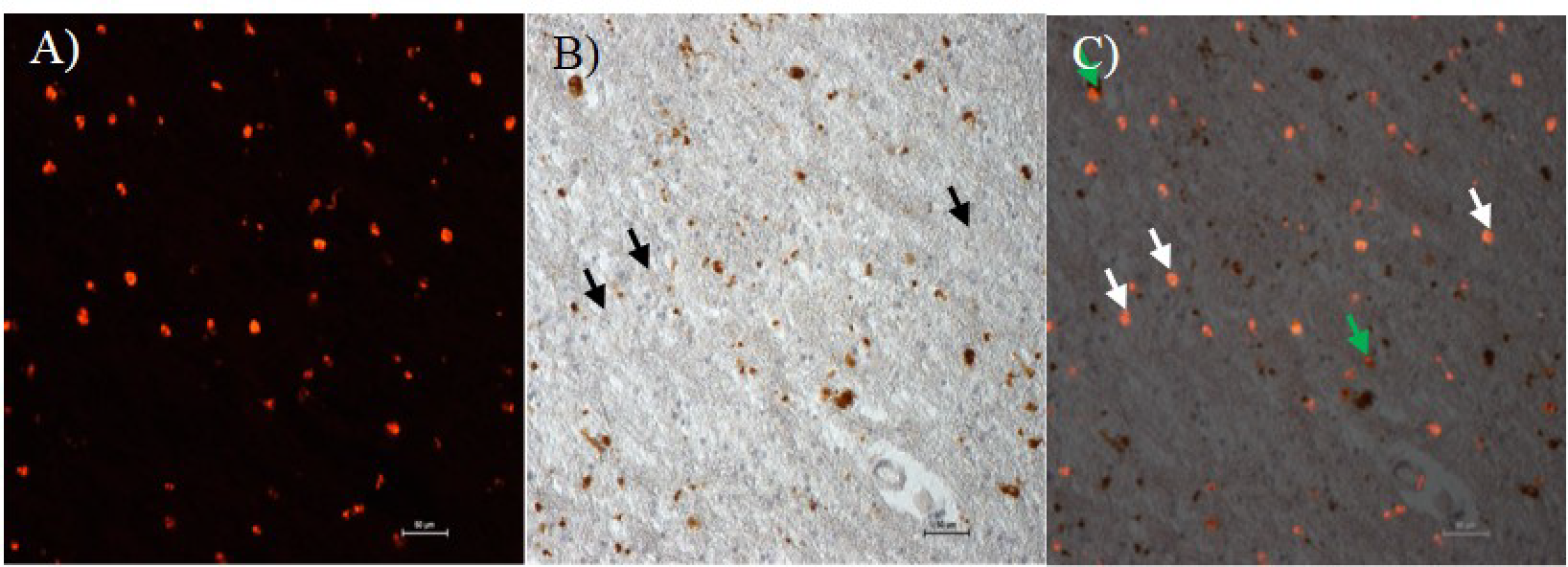

Supplement: S5 Fig — CD68+ (A, red fluorescent label) and Iba-1 detected with 3,3’-diaminobenzidine (DAB) visualised under light microscopy (B, brown). Double labelling confirms colocalisation of CD68 and Iba-1 double-labelled cells (C, green arrows) but also shows a distinct population of Iba-1- cells (B, black arrows) that are CD68+ (C, white arrows) confirming not all CD68+ cells are Iba-1+. Scale bar = 50μm. (TIF) [file pone.0210888.s005.tif]
